# Corticothalamic dynamics during postictal recovery of self-orientation after electroconvulsive therapy

**DOI:** 10.1093/braincomms/fcag144

**Published:** 2026-04-22

**Authors:** Sven Stuiver, Prejaas K B Tewarie, Julia C M Pottkämper, Joey P A J Verdijk, Jeannette Hofmeijer, Guido A van Wingen, Michel J A M van Putten, Jeroen A van Waarde

**Affiliations:** Technical Medical Centre, University of Twente, Enschede 7522 NH, The Netherlands; Department of Psychiatry, Rijnstate Hospital, Arnhem 6815 Ad, The Netherlands; CERVO Brain Research Centre, University of Laval, Québec City, Canada G1J 2G3; Department of Psychiatry, Rijnstate Hospital, Arnhem 6815 Ad, The Netherlands; Department of Psychiatry, Amsterdam UMC Location AMC, Amsterdam 1105 AZ, The Netherlands; Technical Medical Centre, University of Twente, Enschede 7522 NH, The Netherlands; Department of Psychiatry, Amsterdam UMC Location AMC, Amsterdam 1105 AZ, The Netherlands; Technical Medical Centre, University of Twente, Enschede 7522 NH, The Netherlands; Department of Neurology, Rijnstate Hospital, Arnhem 6815 Ad, The Netherlands; Department of Psychiatry, Amsterdam UMC Location AMC, Amsterdam 1105 AZ, The Netherlands; Technical Medical Centre, University of Twente, Enschede 7522 NH, The Netherlands; Department of Neurology and Clinical Neurophysiology, Medisch Spectrum Twente, Enschede 7512 KZ, The Netherlands; Department of Psychiatry, Rijnstate Hospital, Arnhem 6815 Ad, The Netherlands

**Keywords:** EEG, postictal state, corticothalamic model, consciousness, self-orientation

## Abstract

The mechanisms underlying recovery of consciousness after its transient loss remain incompletely understood. Electroconvulsive therapy (ECT) induced generalized seizures disrupt responsiveness and orientation—key functional dimensions related to consciousness—and are followed by a stereotyped postictal state of temporary unresponsiveness, offering a unique model to study the dynamics of connected, report-capable cognition in humans. We performed a *post hoc* analysis of prospectively collected data from a randomized controlled trial, comprising 345 continuous postictal electroencephalography (EEG) recordings of up to 1 h from 33 patients (median age 53 years, interquartile range 21.3 years; *n* = 19 [56%] female) undergoing a course of ECT. Using a corticothalamic mean-field model, we estimated cortical (X), corticothalamic loop (Y) and intrathalamic (Z) gain parameters that quantify the responsiveness of cortical and thalamic populations to synaptic input. We examined whether recovery of self-orientation, indexed by the time to reorientation in person, was associated with specific parameter regimes. Immediately after seizure termination, cortical gain was elevated, the corticothalamic loop gain was negative and intrathalamic gain was near zero—suggesting that the thalamus exerted a suppressive effect on the cortex while exhibiting minimal intrinsic activity. Bayesian mixed-effects models showed that during postictal recovery cortical gain × decreased (*β* = −0.11, CrI_95_ = [−0.16, −0.07]), while corticothalamic loop gain Y (*β* = 0.05, CrI_95_ = [0.02, 0.07]) as well as intrathalamic gain Z (*β* = 0.05, CrI_95_ = [0.02, 0.08]) increased, reflecting progressive restoration of thalamic excitatory drive. Across patients and ECT sessions, recovery of self-orientation occurred when model parameters approached towards a characteristic regime, i.e. *β*_X_ = 0.81 (CrI_95_ = [0.74, 0.88]), *β*_Y_ = −0.11 (CrI_95_ = [−0.16, −0.07]) and *β*_Z_ = 0.01 (CrI95 = [−0.04, 0.05]), for cortical, corticothalamic loop and intrathalamic gains, respectively. No associations were found between model parameters at which self-orientation was regained and reorientation time, showing relatively small inter-subject variability (sd_X_ = 0.04, sd_Y_ = 0.03 and sd_Z_ = 0.02), suggesting that these values were consistent across patients. To conclude, these findings suggest that restoration of effective corticothalamic coupling represents a critical regime for the re-emergence of self-orientation and reportable responsiveness following postictal unresponsiveness. Our results provide time-resolved modelling of human thalamocortical circuit dynamics during postictal recovery, offering mechanistic insight into how large-scale neural interactions reorganize to restore self-orientation during the postictal state.

## Graphical Abstract

**Graphical Abstract:**
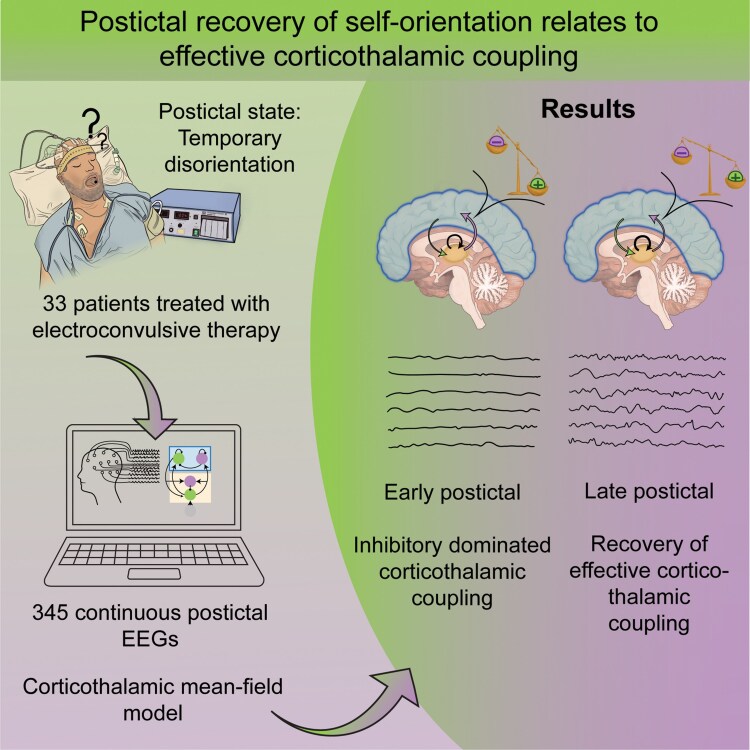

## Teaser

Stuiver *et al*. report that recovery of self-orientation reflects a progressive restoration of effective corticothalamic coupling, providing insights into neurophysiological recovery mechanisms of the postictal state after electroconvulsive therapy.

## Introduction

Consciousness—our capacity for subjective experience and purposeful interaction—presumably emerges from dynamic interactions within and between cortical and subcortical brain systems. However, the circuit-level dynamics that support its breakdown and recovery remain incompletely understood. The mesocircuit hypothesis proposes that conscious awareness depends on a thalamocortical–basal ganglia loop, in which the thalamus plays a key role in maintaining cortical excitability through widespread excitatory projections.^1,2^ Disruption of this circuit is thought to underlie impaired consciousness in conditions such as coma and postictal states, whereas recovery requires reactivation of this thalamocortical drive.^3,4^

Electroconvulsive therapy (ECT) provides a model for studying the recovery of responsiveness and orientation—key functional dimensions related to consciousness—in humans. ECT induces generalized seizures, followed by a reproducible postictal state characterized by transient unresponsiveness and disorientation.^5,6^ During this period, patients gradually regain orientation in person, place and time—clinical markers that serve as proxies for the return responsiveness and orientation, which may be considered functional prerequisites for connected consciousness.^7^ Importantly, the timing and context of ECT are tightly controlled, and the procedure is typically performed under standardized anaesthetic conditions,^8^ providing a controlled setting to study the temporal dynamics of consciousness recovery in a well-defined neural and behavioural context. Although in ECT short-acting anaesthetics (e.g. etomidate) are used, its effects typically resolve within minutes, whereas the recovery of orientation can take 20–30 min, suggesting that the postictal state reflects more than anaesthetic washout alone.^7,9^

Recovery from the postictal state is accompanied by a gradual normalization of electroencephalography (EEG) patterns, including the alpha/delta ratio, typically beginning with generalized suppression, followed by slowing and ultimately a return to baseline activity.^5,7,9,10^ These observations align with the theoretically based ABCD model—which is neurobiologically grounded by the mesocircuit model—and distinguish different spectral regimes in disorders of consciousness (DoC).^11^ The regime transitions are thought to reflect evolving interactions within cortical and subcortical circuits, particularly involving the thalamus. Previous studies have implicated mechanisms such as altered neurotransmitter levels, reduced cortical excitability and transient cerebral hypoperfusion in the postictal state.^5,12-14^ However, how these physiological processes relate to the return of responsiveness and orientation remains unclear.

To address this, we applied a corticothalamic mean-field model to EEG data recorded during the first hour of the postictal period in patients treated with ECT. This model allows estimation of gain parameters that reflect the responsiveness of cortical and thalamic neuronal populations to synaptic input, offering a window into the evolving dynamics of the thalamocortical system during sleep arousal states and seizures.^15,16^ In a previous study, we showed that during seizure termination, just before entering the postictal period, cortical excitation and corticothalamic drive increase, while intrathalamic strength diminishes.^17^

Here, we studied how these corticothalamic gain parameters evolve after seizure termination and how these relate to the behavioural recovery of self-orientation as a functional marker supporting the re-emergence of connected consciousness. We hypothesized that the re-emergence of self-orientation is associated with a dynamic rebalancing of cortical, corticothalamic and intrathalamic gain, as predicted by the mesocircuit hypothesis.

## Materials and methods

### Study population

This was a *post hoc* analysis of prospectively collected EEG data from 33 patients treated with ECT for treatment-resistant major depressive disorder in Rijnstate Hospital (Arnhem, The Netherlands). These patients participated in the StudY of the effect of Nimodipine and Acetaminophen on Postictal Symptoms after ECT (SYNAPSE; NCT04028596) and were aged 18 years or older and indicated for ECT due to major depression [classified as unipolar, bipolar, or schizoaffective disorder, according to the Diagnostic Manual of Mental Disorders, fifth edition (DSM-5)].^18^ The study protocol was approved by the local medical ethics committee, and all patients provided oral and written informed consent, according to the Declaration of Helsinki. More details are provided in the published study protocol and original publication.^19,20^

### Electroconvulsive therapy

ECT was administered twice a week, following the standard treatment guidelines in the Netherlands.^21^ Depending on clinical indication, unilateral (UL)^22^ or bi(fronto)temporal (BL) placements of the ECT electrodes were used. Stimulation was delivered using a Thymatron System IV (Somatics, Lake Bluff, IL, USA), providing brief, bidirectional, square-wave pulses (1.0 ms duration) at a constant current of 0.9 A. The stimulus dose was determined using the age-based method for BL and titration-based dosing for RUL treatments, following local protocol. All patients were treated under general anaesthesia, typically with intravenous etomidate (0.15–0.3 mg/kg) and received muscle relaxation with succinylcholine (0.5–1.0 mg/kg) to prevent motor convulsions. Hyperventilation was routinely applied to optimize seizure duration. Hamilton Depression Rating Scale scores were assessed before and after the treatment course, in line with clinical routine. Clinical outcomes were not analysed in relation to EEG parameters in the current study.

### Recovery of self-orientation

The reorientation time (ROT) questionnaire was used to assess recovery of self-orientation and basic responsiveness, rather than recovery of consciousness *per se*.^23^ It consists of five questions (asked at an interval of 5 min, started from induction of the electrical stimulus) to determine the timepoints of recovery in the cognitive domains of person, place and time. Specifically, we used the ROT in person, which assesses the ability to correctly identify oneself and retrieve autobiographical semantic information (e.g. name and date of birth). This measure reflects the recovery of personal orientation and basic responsiveness, rather than recovery of conscious experience or full environmental connectedness. Throughout the manuscript, we therefore use the term ‘self-orientation’ to denote the specific functional domain captured by ROT in person.

### EEG registration and pre-processing

Twenty silver/silver chloride cup electrodes were placed on the scalp according to the International 10–20 system. Sometimes, a reduced montage of 12 electrodes was used for feasibility. Cz was used as a reference. The electrode-skin impedance was kept below 5 kΩ. Baseline EEG recordings of 5 min with eyes closed were made prior to the start of the ECT course. Continuous postictal EEG recordings up to 1 h after ECT stimulus administration were made up to the 13th ECT session. EEGs were recorded with a DC amplifier at a sampling frequency of 256 Hz (NeuroCenter EEG, Leiden, the Netherlands). All analyses were performed offline. After band-pass filtering (0.5–45 Hz; first-order Butterworth filter), EEG recordings were visually inspected for artefacts. Electrodes containing noise or electrodes that became detached during the measurements (e.g. due to patient movement) were rejected for analyses. All EEG analyses, pre-processing, parameter estimations and model fitting were conducted with MATLAB R2023a (MathWorks, Natick, MA, USA).

### Corticothalamic mean-field model

**Figure 1:**
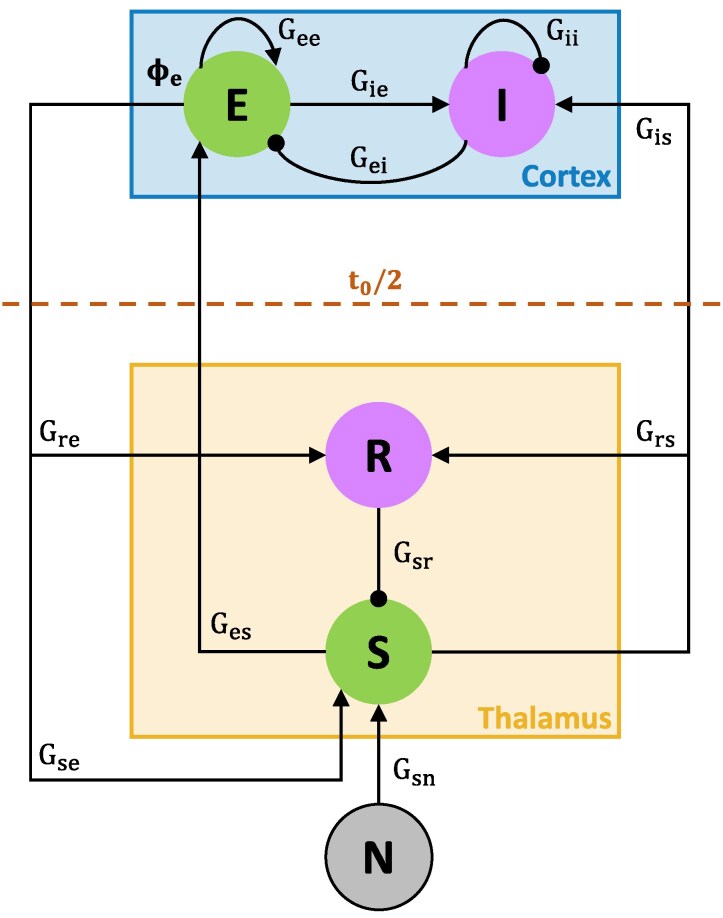
**Schematic overview of the Robinson corticothalamic mean-field model.** The model consists of two cortical [excitatory (E) and inhibitory (I)] and two thalamic [reticular (R) and relay (S)] populations and a non-corticothalamic population (N). Here, the interactions between populations are indicated by gain parameters that represent the effective synaptic coupling strengths of a neural population to its synaptic inputs. Gains from population *b* to population *a* are shown as *G*_ab_. The model’s electroencephalogram (EEG) is generated by the dynamics of the excitatory population *ϕ*_e_. Signals propagating from the thalamus to the cortex are delayed by *t*_0_/2 (i.e. 40 ms). Arrowheads and dotheads represent excitatory and inhibitory interactions, respectively. Figure with permission from the original publication.^17^.

We employed the corticothalamic mean-field model of Robinson *et al.*^14^ (Fig. 1), consisting of four interacting neural populations: two cortical populations [excitatory (*E*) and inhibitory (*I*)] and two thalamic populations [relay (*R*) and reticular (*S*)]. The model operates in a cycle through three steps: (i) converting the mean membrane potential *V*_a_ of each population into a firing rate *Q*_a_ using a sigmoid function, (ii) propagating the firing rate *ϕ*_a_ through the neural tissue via a damped wave equation and (iii) incorporating this damped firing rate on a target population (either increasing or decreasing its potentiation). Each population has a mean membrane potential *V*_a_, where a ∈{*e*, *i*, *r*, *s*}, which is converted to a firing rate *Q*_a_ by a sigmoid function, given by:


(1) $$Q_{a}=\frac{Q_{max}}{1+\exp\left(-\frac{V_{a}-\theta }{\sigma^{\prime }}\right)}$$

where $Q_{max}$ is the maximum firing rate, *θ* is the mean threshold potential and *σ*^′^ represents the standard deviation of this threshold (set as *π*/3). The potential *V*_a_(t) depends on the incoming firing rate *ϕ*_a_ (t) from other populations and/or itself using


(2) $$\left(\frac{1}{\alpha \beta }\frac{d^{2}}{dt^{2}}+\left(\frac{1}{\alpha }+\frac{1}{\beta }\right)\frac{d}{dt}+1\right)V_{a}(t)=\underset{a^{\prime }}{\sum }\;v_{aa^{\prime }}\phi_{a^{\prime }}(t)+\underset{b}{\sum }\;v_{ab}\phi_{b}(t-t_{0}/2).$$

Here, *α* and *β* are the synaptic rise and decay constants, respectively, $v_{aa^{\prime }}$ and $v_{ab}$ represent the synaptic strength between populations and *t*_0_ is the propagation delay (time constant) between thalamic and cortical populations. The mean firing rate *ϕ*_a_(t) is temporally damped to account for dendritic effects using


(3) $$\left(\frac{1}{\gamma_{a}^{2}}\frac{d^{2}}{dt^{2}}+\frac{1}{\gamma_{a}}\frac{d}{dt}+1\right)\phi_{a}(t)=Q_{a}(t)$$

where *γ*_a_ is the temporal damping rate (*γ* = *v*_a_/*r*_a_), with *v*_a_ the propagation velocity and *r*_a_ is the mean axonal range. According to the local inhibition approximation,^24^  *γ*_a_ ≈ 0 for the inhibitory, relay and reticular populations, hence simplifying *ϕ*_a_(*t*) = *Q*_a_(*t*) for these populations.

### Model parameter estimation

Temporal evolutions of the biophysical model gain parameters were estimated from postictal EEG power spectral densities (PSDs) using the Metropolis–Hastings algorithm. This Markov chain Monte Carlo (MCMC) random walk method generates the probability distribution for each model parameter. For each EEG channel, PSDs of artefact-free segments of 5 s were calculated using Welch’s method.^25^ For the baseline EEG, an averaged PSD was calculated over the entire measurement, and in the case of the postictal state, PSD values were averaged per minute. The median PSD value was computed across the available electrode channels to account for inter-channel variability. In this corticothalamic mean-field model, the EEG is generated by the dynamics of the cortical excitatory population (*ϕ*_e_) and its power spectrum is obtained by taking the squared magnitude of the Fourier transform of *ϕ*_e_(*t*). Details of parameter estimation and fitting steps are described elsewhere.^16^ We employed the corticothalamic mean-field model to estimate five biophysical gain parameters. Model gain parameters quantify the responsiveness of a neural population to its inputs. Essentially, a gain parameter is the product of the sensitivity of the firing rate to voltage changes and the coupling strength.^15^ It is given by:


(4) $$G_{ab}\;=\;S^{\prime }(Va(t))\mu_{ab},$$

where $S^{\prime }(V_{a}(t))=\frac{dS(V)}{dV}|{\;}_{V=V_{a}(t)}$ is the instantaneous slope (sensitivity) of the sigmoid at the current membrane potential $V_{a}(t)$ and $\mu_{ab}(t)$ is the coupling strength from population *b* to *a*. Note that the neural mass is most excitable when its steady state voltage equals its mean threshold, corresponding to the point of the maximal slope of the sigmoid. Further details of gain parameters are provided in the Supplementary Gain parameters. The gain parameter *X* reflects the net excitatory–inhibitory (*E*/*I*) balance within the cortex, *Y* reflects the overall feedback in the corticothalamic loop and *Z* indicates the thalamus’ intrinsic responsiveness. These gain parameters are given by:


(5) $$X=\frac{G_{ee}}{1-G_{ei}},\;Y=\frac{G_{ese}+G_{esre}}{\left(1-G_{srs})(1-G_{ei}\right)},\;Z=-G_{srs}\frac{\alpha \beta }{{\left(\alpha +\beta \right)}^{2}}.$$

Subsequently, we calculated partial corticothalamic (*G*_ct_) and thalamocortical (*G*_tc_) gains, which we defined as:


(6) $$G_{ct}=\frac{G_{ese}}{\left(1-G_{srs})(1-G_{ei}\right)},\;G_{tc}=\frac{G_{esre}}{\left(1-G_{srs})(1-G_{ei}\right)}$$

where *Y* = *G*_ct_ + *G*_tc_. Parameter values for parameters were constrained to remain within neurophysiologically plausible ranges.^15^ Model parameters were estimated by iteratively optimizing the fit between the averaged observed and model’s power spectrum for each minute (i.e. 40–60 min) during the postictal state. For the baseline EEG, averaged model parameters were calculated from a single, averaged baseline PSD. Chi-square (*χ*^2^) goodness-of-fit tests were performed to determine the quality of the fits (where *χ*2 = 0 implies a perfect fit).

### Fitting temporal evolutions of model parameters

A saturating exponential decay, a saturating exponential growth and a sigmoidal fit were applied to each model parameter trajectory to characterize its temporal evolution during the postictal state. The best model fit was selected using a custom-written algorithm based on the Akaike information criterion (AIC).^26^ The fitting model with the lowest AIC was considered the one best fitting the data using the fewest possible independent variables. A difference in AIC larger than two was considered significantly different. Finally, the difference between gain values at *t* = 0 and *t* = 60 was determined from the best model fit and used as a measure of the temporal change.

### Statistical analysis

#### Temporal changes in model parameters

To characterize the temporal behaviour of model parameters, we fitted two Bayesian multivariate generalized linear mixed models (random intercepts and slopes for ECT session across subjects) using MCMC sampling to account for the repeated-measures structure of the data and the potential correlations between outcome variables (i.e. model gain parameters). The first model included the overall gain parameters *X*, *Y* and *Z*, while the second focused on partial gain parameters: cortical excitatory feedback (*G*_ee_), cortical inhibitory feedback (*G*_ei_), corticothalamic gain (*G*_ct_), thalamocortical gain (*G*_tc_) and thalamic inhibitory feedback (*G*_srs_). This approach allowed us to study both generalized and specific effects of gain parameters. The temporal change over the first 60 min postictally (i.e. the temporal evolution described above) was used as the response variable. These models were used to assess whether and how the gain parameters changed during the first 60 min postictally (i.e. their temporal evolution).

#### Recovery of self-orientation and model parameter regimes

To examine whether recovery of self-orientation occurred when the model parameters (*X*, *Y* and *Z*) reached a specific regime, we analysed the association between the model parameters and ROT in person. For each measurement, we determined the estimated model parameter values from the best model fits corresponding to three time points (i) the immediate postictal state (i.e. the first minute), (ii) the ROT time point (i.e. when a patient was first reoriented in person) and (iii) the late postictal state (i.e. at 60 min). For each of these three states of consciousness, a Bayesian linear mixed effects model with random intercepts and slopes for ECT session across subjects was fitted with model parameter gains (i.e. *X*, *Y* and *Z*) as outcome parameters. Fixed effects included ECT parameters (seizure duration, electrode placement, charge, etomidate dose, postictal administration of benzodiazepine and ECT session number). We used the brms package for Bayesian regression modelling in R version 4.3.1.^27-29^ Default weakly informative priors were applied to enhance numerical stability while minimizing prior influence. Specifically, flat (improper) priors were used for fixed effects and weakly informative priors for random effects and residual standard deviations. Gaussian likelihoods were assumed for all response variables. Models were estimated using the default MCMC settings: four chains, each with 2000 iterations, including 1000 warm-up iterations, resulting in 4000 post-warm-up samples for inference. Convergence was assessed using Rhat statistics (all ∼1.00), and model fit was evaluated via posterior predictive checks. Results were reported as posterior means with 95% credible intervals.

## Results

**Figure 2:**
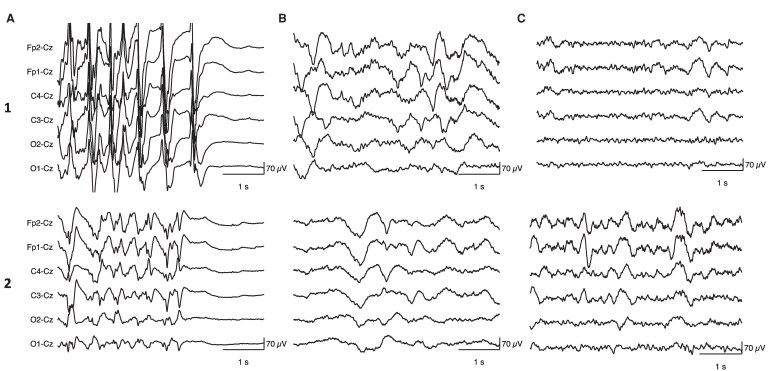
**Examples of typical electroencephalogram (EEG) evolutions during the first hour of the postictal state.** Directly after seizure termination, the EEG shows abrupt suppression (**A**). During the early postictal state (i.e. 10 min after seizure termination), slow-wave activity dominates the EEG (**B**). Recovery of background activity after 1 h (**C**). Numbers 1 and 2 refer to Patients 1 and 2 of the study, respectively.

We included a total of 345 ECT-induced postictal EEGs from 33 patients. The patients’ median age was 53 years (IQR 21.3), 19 patients were female (56%) and most patients were treated with BL ECT (73%, *n* = 24). On average, patients received anaesthesia with 20.1 ± 4.0 mg etomidate. Patients self-orientated (i.e. ROT in person) on average after 22.4 ± 4.8 min (mean ± SD). Half of the patients (50%) showed clinical antidepressive response, and 22% reached remission of depression after the ECT course. A reduced montage was used in 145 (42%) of the included postictal EEG recordings. For more details about patient and treatment characteristics, we refer to the original study.^19^ Fig. 2 shows an example of two typical EEG evolutions from the early postictal state (i.e. directly after seizure termination) to the late postictal state (i.e. after 1 h). Results of the fitting models that were employed to determine the temporal evolutions of the model parameters and fitting performances of all models are shown in Supplementary Table 1. All estimated model parameters showed convergence, with Rhat values of 1.00. Posterior predictive checks and goodness-of-fit values of model parameter estimations are provided in Supplementary Figs 1 and 2, respectively.

### Dynamic rebalancing of cortical and corticothalamic gains during postictal recovery

**Figure 3:**
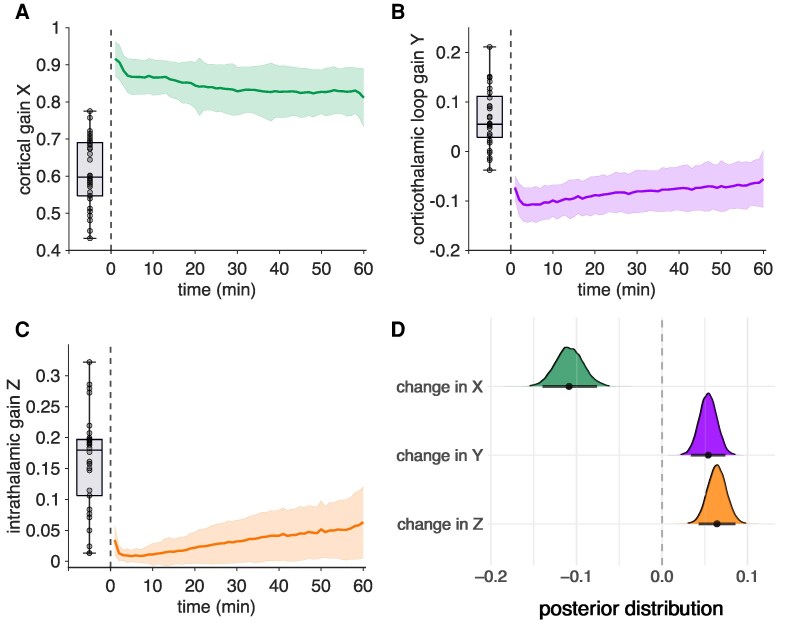
**Cortical gain decreases, whereas corticothalamic loop and intrathalamic gain increase during the first hour of the postictal state.** Mean postictal values across patients and electroconvulsive therapy (ECT)-sessions of cortical gain *X* (**A**), corticothalamic loop gain *Y* (**B**) and intrathalamic gain *Z* (**C**) are presented for illustrative purposes. Posterior distributions with 95% credible intervals indicate that *X* decreased, while *Y* and *Z* increased during postictal recovery (**D**). Solid lines and shaded areas represent means and standard deviations, respectively. Boxplots show the baseline gain values prior to the ECT course. The posterior estimates in D are derived from a Bayesian multivariate mixed-effects model with subject-level random effects, based on *n* = 345 postictal electroencephalography (EEG) recordings from *n* = 33 patients.

Directly after seizure termination (i.e. in the early postictal state), the cortical gain *X* was close to 1, the corticothalamic loop gain *Y* was negative and the intrathalamic gain *Z* was close to 0 (Fig. 3A–C). The initial postictal values showed deviations from baseline. These findings suggest that cortical E/I ratios are increased, the activity within the corticothalamic loop is inhibitory-dominated and that the thalamus is almost completely insensitive for incoming inputs during the first period of the postictal state. Bayesian multivariate analysis showed that cortical gain *X* decreased (*β* = −0.11, CrI_95_ = [−0.16, −0.07]), while corticothalamic loop gain *Y* (*β* = 0.05, CrI_95_ = [0.02, 0.07]) as well as intrathalamic gain *Z* (*β* = 0.05, CrI_95_ = [0.02, 0.08]) increased during the first hour of the postictal state. These findings suggest a decrease in cortical *E*/*I* ratio, an increase of excitatory activity in the corticothalamic loop and an increase in thalamic sensitivity to incoming inputs during the first postictal hour. All parameters changed towards baseline values prior to the ECT course, but did not reach baseline values within 60 min. Posterior distributions of the multivariate model with 95% credible intervals are shown in Fig. 3D.

### Partial cortical, corticothalamic and thalamic gains undergo dynamic reorganization during the postictal state

**Figure 4:**
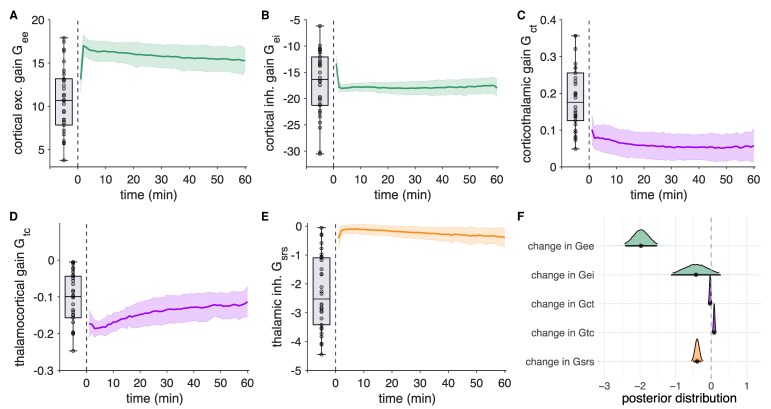
**Temporal evolutions of corticothalamic mean-field model (partial gain) parameters during the first hour of the postictal state.** Mean postictal values across patients and electroconvulsive therapy (ECT)-sessions of cortical excitatory *G*_ee_ (**A**), cortical inhibitory *G*_ei_ (**B**), corticothalamic *G*_ct_ (**C**), thalamocortical *G*_tc_ (**D**) and thalamic inhibitory *G*_srs_ (**E**) gains are presented for illustrative purposes. Posterior distributions with 95% credible intervals indicate that *G*_ee_, *G*_ct_ and *G*_srs_ decreased, while *G*_tc_ increased during postictal recovery (**F**). Solid lines and shaded areas represent means and standard deviations, respectively. Boxplots show the baseline gain values prior to the ECT course. The posterior estimates in F are derived from a Bayesian multivariate mixed-effects model with subject-level random effects, based on *n* = 345 postictal electroencephalography (EEG) recordings from *n* = 33 patients.

Immediately after seizure termination, we did not observe deviations from baseline for the cortical excitatory *G*_ee_, the inhibitory *G*_ei_, nor the thalamocortical *G*_tc_ gain (Fig. 4A, B and D). In contrast, the corticothalamic *G*_ct_ gain appeared decreased and thalamic inhibitory feedback gain *G*_srs_ increased, compared to baseline values (Fig. 4C and E). Bayesian multivariate analysis showed that *G*_ee_ (*β* = −1.97, CrI_95_ = [−2.59, −1.37]), *G*_ct_ (*β* = −0.03, CrI_95_ = [−0.06, −0.00]) and *G*_srs_ (*β* = −0.34, CrI_95_ = [−0.51, −0.18]) decreased during the first hour of postictal recovery. On the other hand, *G*_tc_ increased (*β* = 0.08, CrI_95_ = [0.05, 0.11]). The posterior estimate of *G*_ei_ (*β* = −0.59, CrI_95_ = [−1.56, 0.37]) indicated a possible decrease, but with substantial uncertainty. Posterior distributions with 95% credible intervals of the multivariate model are shown in Fig. 4F.

### Recovery of self-orientation emerges within a characteristic model parameter regime

**Figure 5:**
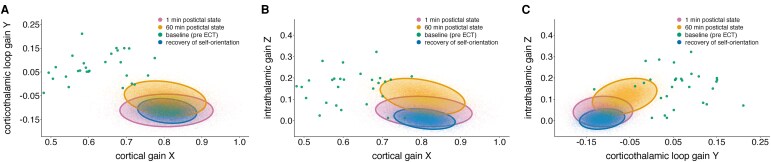
**Recovery of self-orientation occurs within a specific corticothalamic gain parameter regime during postictal recovery.** Joint posterior distributions (95% credible ellipses) of the model-derived gain parameters (i.e. cortical *X*, corticothalamic loop *Y* and intrathalamic *Z* gains) at the first minute of the postictal state (in purple), at the time of recovery (on average 22.4 ± 4.8 min) of self-orientation (in blue) and at 60 min of the postictal state (in orange). The majority of the model parameter values deviate from baseline values before the electroconvulsive therapy (ECT) course (in green). Despite clear electrophysiological changes from early postictal suppression to restored cortical activity, the gain parameter regimes remain overlapping, implying that recovery reflects reorganization of connectivity within a stable dynamical regime rather than a shift to a new one. This figure depicts posterior estimates derived from Bayesian multivariate mixed-effects models with subject-level random effects, based on *n* = 345 postictal electroencephalography (EEG) recordings from *n* = 33 patients. Each panel (**A–C**) shows a different pairwise projection of the multivariate parameter space.

Across ECT sessions and patients, recovery of self-orientation occurred when model parameters approached *β*_X_ = 0.81 (CrI_95_ = [0.74, 0.88]), *β*_Y_ = −0.11 (CrI_95_ = [−0.16, −0.07]) and *β*_Z_ = 0.01 (CrI_95_ = [−0.04, 0.05]), for cortical, corticothalamic loop and intrathalamic gains, respectively. Figure 5 shows the joint parameter distributions of these parameters at the time of recovery of self-orientation, alongside those obtained during the first minute of the postictal state (unresponsive) and after 60 min of postictal recovery. The joint parameter regimes exhibited substantial overlap, indicating that recovery of self-orientation reflects a gradual convergence towards a characteristic region of parameter space rather than a discrete shift between distinct regimes.

We did not find any associations between the model parameter values at which self-orientation was regained and the ROT value. In other words, a shorter or longer time to reorientation in person was not associated with higher or lower model parameter values. Therefore, recovery of self-orientation may coincide with similar model parameter values, regardless of the time point at which this occurs. The inter-subject variability was relatively small (sd_X_ = 0.04, sd_Y_ = 0.03 and sd_Z_ = 0.02) for all model parameters, suggesting that these values were consistent across patients. No relationships were found between model parameter values and the ECT parameters and etomidate dose (*β_X_* = −0.00, CrI_95_ = [−0.01, 0.00], *β_Y_* = −0.00, CrI_95_ = [−0.00, 0.00] and *β_Z_* = −0.00, CrI_95_ = [−0.00, 0.00]). Regarding partial gain parameters, cortical excitatory gain *G*_ee_ (*β* = 0.07, CrI_95_ = [0.02, 0.12]) and partial thalamic inhibitory gain *G*_srs_ (*β* = 0.02, CrI_95_ = [0.01, 0.03]) increased over the ECT course. These associations suggest that patients who were further along the ECT course (i.e. who had more previous ECT seizures) showed less postictal decrease in *G*_ee_ and *G*_srs_.

## Discussion

The ability to be conscious—with an accurate orientation in the world, in time and in ourselves—makes us human. Mechanisms of recovery of conscious interaction with the environment after its transient loss (e.g. due to seizure, head trauma, intoxication) remain incompletely understood. We studied the corticothalamic dynamics involved in the recovery of self-orientation—a key dimension related to consciousness—after ECT-induced seizures using continuous EEG recordings and a biophysically grounded mean-field model. We show that recovery of self-orientation reflects a progressive restoration of thalamocortical dynamics, characterized by changes in cortical and thalamic gain parameters. Moreover, recovery consistently occurred within a characteristic region of model parameter space, suggesting an involvement of a specific corticothalamic operating regime.

### Dynamic corticothalamic re-engagement as candidate mechanism for recovery of self-orientation

Immediately after seizure termination, the cortical gain *X* was near maximal, the activity in the corticothalamic loop gain *Y* was dominated by inhibition and the intrathalamic gain *Z* was near zero. These findings reflect a state of functional disconnection between the thalamus and cortex. Over the first hour of postictal recovery, cortical gain gradually declined, while both corticothalamic loop and intrathalamic gains increased, suggesting progressive re-engagement of thalamic input. Previously, we showed that early postictal suppression is marked by strongly negative normalized alpha/delta ratios, which increase during recovery.^7^ Therefore, the corticothalamic gain parameters we studied here may underlie this transition. Analysis of partial gain parameters indicated that these changes were primarily driven by a reduction in cortical excitatory gain, an increase in thalamocortical gain (that outweighed a concurrent decrease in corticothalamic feedback) and an increase in thalamic inhibitory gain. Notably, patients with more previous ECT sessions showed less postictal decrease in both excitatory cortical and inhibitory thalamic gain. These findings point towards a dynamic corticothalamic re-engagement as a candidate mechanism for early postictal recovery of self-orientation.

#### Functionally disconnected cortex due to reduced thalamic input and impaired synaptic feedback

The cortical gain *X* values close to 1 in the immediate postictal state indicate that the cortex was highly responsive to its own inputs. This may appear contradictory, as postictal EEG is typically characterized by suppression and slowing.^5,6,10^ However, the gain reflects the sensitivity to input—i.e. effective synaptic coupling—rather than actual firing rates or activity levels. Mathematically, it is defined as the product of the instantaneous slope of the sigmoid function (*S*^′^(*V*_a_)) at the membrane potential (*V*_a_(*t*)) and the synaptic coupling strength (*µ*_ab_) (Eq. 4). The slope *S*^′^(*V*) is maximal when the operating point lies near the threshold (*V* ≈ *θ*) and a high *µ*_ab_ further amplifies the gain. Importantly, these two mechanisms can interact: a large *µ*_ab_ may still yield a high gain even when the operating point is suboptimal and vice versa. This distinction is essential for interpreting postictal states, where suppressed EEG activity may arise either from intrinsically reduced excitability or from functional disconnection due to weakened synaptic feedback. If the gain is low despite the operating point being near the maximal slope of *S*(*V*_a_), this suggests that synaptic coupling strength *µ*_ab_ is reduced. In such cases, the neural population may still be excitable in principle but is functionally decoupled due to insufficient feedback.

The strongly negative corticothalamic loop gain *Y* indicates a net inhibitory effect from the thalamus to the cortex. This suggests a functional decoupling, with thalamic inhibition actively suppressing cortical excitability. Consistent with this, the model did not support the generation of alpha rhythms, which typically require *Y* > 0 and instead predicted dominance of delta and theta activity—matching EEG observations, in line with clinical observations.^5,6,10^ At the same time, intrathalamic gain *Z* was close to zero, implying that the thalamus exhibited minimal intrinsic responsiveness. This could arise from a shift in the operating point away from the region of maximal excitability, reduced synaptic efficacy, or a temporary lack of input from the cortex. Whether due to intrinsic suppression or cortical disengagement, the thalamus appeared functionally silent in this early stage, effectively disconnecting the cortex from its normal excitatory thalamic drive. This transient disconnection likely serves a protective role—reducing cortical excitability to prevent immediate reactivation or propagation of seizure activity.^30,31^ Together, these observations suggest that in the early unconscious state, the cortex is excitable in principle, but functionally disconnected due to reduced thalamic input and impaired feedback. As patients regain connected, report-capable responsiveness and become re-oriented to person, place and time (usually within 1 h),^7^ both the cortical excitatory gain *G*_ee_ and the thalamic inhibitory gain *G*_srs_ typically decrease, indicating a normalization of neural responsiveness.

#### Early postictal hyperexcitability may be driven by transient hypoxia

A plausible biological mechanism involved in the apparent initial and transient high cortical gain *X*, reflecting an increased potential for excitatory response, relates to postictal hypoperfusion, which is associated with selective changes in synaptic transmission.^32^ Temporary hypoxia has been observed in the postictal state in both humans and animal models,^13,14,33^ and has been related to increased cortical excitability.^34,35^ Elsewhere, we reported reduced (and increased) global and regional postictal cerebral blood flow, in the same study cohort measured with arterial spin labelling magnetic resonance imaging.^33^ Although excitatory neurons are affected by hypoxia, inhibitory interneurons are often more vulnerable, leading to a loss of inhibition and a net increase in cortical excitability,^36^ which may explain the initially increased cortical excitatory *G*_ee_ and unaffected cortical inhibitory *G*_ei_ values. As the cortex recovers, it becomes less sensitive to excitatory inputs, supporting the hypothesis that early postictal hyperexcitability may be driven by transient hypoxia.

#### Model-based insight into evolving postictal corticothalamic responsiveness may offer a framework for personalized treatment of disorders of consciousness

Our findings support the mesocircuit hypothesis that the thalamus is an essential relay hub, strongly interconnected with the cortex, essential for self-orientation—a key dimension related to consciousness.^1^ While the excitatory output from the thalamus to the cortex (*G*_tc_) increased during postictal recovery, the influence of the cortex on the thalamus (*G*_ct_) decreased. Thus, as the brain recovers from the ECT seizure, postictal recovery may involve an increase in the excitatory input from the thalamus to the cortex. While prior studies have highlighted the importance of thalamocortical excitation for recovery of consciousness,^3,4^ these were largely descriptive or based on imaging modalities with limited temporal resolution. In contrast, our model-based approach provides time-resolved, mechanistic insight into the evolving synaptic responsiveness of the corticothalamic system during postictal recovery in humans. Beyond providing insights into recovery dynamics, these mechanistic findings may inform potential treatment strategies. Recently, an *in silico* study demonstrated that mathematically derived stimulation protocols could induce healthy-like neural activity patterns using the same model, offering a potential framework for personalized treatment of disorders of consciousness.^37^

#### Critical operating state of thalamocortical dynamics required for self-orientation

Furthermore, we identified a characteristic regime of model-derived corticothalamic gain parameters at the time of recovery of orientation in a person. This regime suggests the existence of a critical operating state of thalamocortical dynamics required for the re-emergence of self-orientation after ECT-induced seizures. Notably, this regime appeared independent of the time required to reach it, implying that recovery depends more on recovery of corticothalamic dynamics rather than on the elapsed time since seizure termination. Together, these findings suggest that recovery of self-orientation does not depend on a gradual linear change in model parameters *per se*, but rather on the system’s return to a specific and reproducible region of corticothalamic parameter space. The consistency of this parameter regime across our individual patients implies the existence of a shared dynamical mechanism underlying the restoration of self-orientation after generalized seizures. This framework may help explain inter-individual differences in recovery times (which is often seen in clinical ECT practice, independent of antidepressive efficacy or cognitive side-effects).

#### ROT as measure for recovery of self-orientation

Our study used orientation in person from the ROT questionnaire (which appeared on average 22.4 ± 4.8 min) as a time-locked, practical measure of early postictal cognitive recovery. ROT captures recovery of self-orientation and basic responsiveness, which are functional dimensions necessary for connected consciousness. However, it does not directly assess the subjective experience. While the mesocircuit hypothesis focuses on neural conditions enabling consciousness awareness rather than on ROT itself, the corticothalamic dynamics estimated in our study may share a link between these functional capacities. Orientation in person is one of the earliest cognitive functions to return and reflects basic arousal and awareness, yet it remains an indirect behavioural measure. It therefore reflects a bundle of capacities that extend beyond the mere presence of conscious experience, which itself can be present without connectedness or responsiveness (e.g. disconnected consciousness).^38^ Relatedly, behaviourally unresponsive patients may still perform cognitive tasks.^39^ Nevertheless, by linking ROT to time-resolved electrophysiological measures, our study offers mechanistic insights into how corticothalamic dynamics evolve to support early return of self-orientation after generalized seizures.

This study has some limitations. Spatial differences in the EEG measures were ignored, as we averaged the power spectra across all EEG electrodes. Although the first period of the postictal state shows limited spatial variation, differences may develop over time; whether these are clinically relevant or physiologically meaningful remains to be determined. However, including more parameters may complicate interpretability and overfitting. Another limitation was that our patients used concomitant medications (including antipsychotics, antidepressants, and benzodiazepines, dosages kept constant during the study), which may have influenced the EEG. Finally, ECT was administered under general anaesthesia using etomidate, which may also have affected the early postictal EEG and the estimates of initial postictal model parameters. However, the postictal evolution of the alpha/delta ratio is very similar in ECT patients compared with epilepsy—and who do not receive etomidate—suggesting limited effects of etomidate on postictal EEG.^8^ Furthermore, the concentration of etomidate in the brain declines very quickly—within minutes—after administration, suggesting that these effects may only have been present in the first minutes of the postictal state.^40^ Finally, patients typically began to regain orientation within 30–60 min after ECT, and our results showed that the etomidate dose did not affect model parameters and ROT values. While we cannot fully exclude early contributions of etomidate to the suppression of cortical or thalamic activity, the progressive and time-locked evolution of model parameters during recovery strongly suggests a leading role for seizure-induced network reorganization.

## Conclusion

Our study reveals key features of the temporal dynamics underlying recovery of self-orientation following ECT-induced seizures. Immediately after seizure termination, the cortex is functionally decoupled from its normal thalamic drive due to strong thalamic inhibition. As recovery progresses, we observe a gradual decrease in the cortical excitation-inhibition ratio, reactivation of the corticothalamic loop and restoration of intrinsic thalamic responsiveness. Recovery of self-orientation occurred in a characteristic region of the parameter space, indicating a stable re-establishment of corticothalamic operating dynamics. These findings offer new insights into the neurophysiological mechanisms supporting the transition from unresponsiveness to orientation in the postictal state.

## Supplementary Material

fcag144_Supplementary_Data

## Data Availability

Raw data were generated at Rijnstate. Derived and anonymized data supporting the findings of this study are available from the corresponding author on request. The code for the parameter estimation method can be found on https://github.com/BrainDynamicsUSYD/braintrak.

## References

[1] Schiff ND . Recovery of consciousness after brain injury: A mesocircuit hypothesis. Trends Neurosci. 2010;33:1–9. 19954851 10.1016/j.tins.2009.11.002 PMC2931585

[2] Schiff ND . Cognitive motor dissociation following severe brain injuries. JAMA Neurol. 2015;72:1413–1415. 26502348 10.1001/jamaneurol.2015.2899

[3] Edlow BL, Claassen J, Schiff ND, Greer DM. Recovery from disorders of consciousness: Mechanisms, prognosis and emerging therapies. Nat Rev Neurol. 2021;17:135–156. 33318675 10.1038/s41582-020-00428-x PMC7734616

[4] Huang Z, Mashour GA, Hudetz AG. Propofol disrupts the functional core-matrix architecture of the thalamus in humans. Nat Commun. 2024;15:1–13. 39251579 10.1038/s41467-024-51837-1 PMC11384736

[5] Fisher RS, Engel JJ. Definition of the postictal state: When does it start and end? Epilepsy Behav. 2010;19:100–104. 20692877 10.1016/j.yebeh.2010.06.038

[6] Pottkämper JCM, Hofmeijer J, van Waarde JA, van Putten MJAM. The postictal state—What do we know? Epilepsia. 2020;61:1045–1061. 32396219 10.1111/epi.16519 PMC7317965

[7] Stuiver S, Pottkämper JCM, Verdijk JPAJ, et al Restoration of postictal cortical activity after electroconvulsive therapy relates to recovery of orientation in person, place and time. Eur Psychiatry. 2024;67:1–7. 38351599 10.1192/j.eurpsy.2024.10 PMC10966617

[8] Pottkämper JCM, Verdijk JPAJ, Hofmeijer J, van Waarde JA, van Putten MJAM. Seizures induced in electroconvulsive therapy as a human epilepsy model: A comparative case study. Epilepsia Open. 2021;6:672–684. 34351710 10.1002/epi4.12532 PMC8633469

[9] Pottkämper JCM, Verdijk JPAJ, Stuiver S, et al Seizure duration predicts postictal electroencephalographic recovery after electroconvulsive therapy-induced seizures. Clin Neurophysiol. 2023;148:1–8. 36773503 10.1016/j.clinph.2023.01.008

[10] So NK, Blume WT. The postictal EEG. Epilepsy Behav. 2010;19:121–126. 20724219 10.1016/j.yebeh.2010.06.033

[11] Schiff ND . Mesocircuit mechanisms underlying recovery of consciousness following severe brain injuries: Model and predictions. In: Brain function and responsiveness in disorders of consciousness. Springer; 2016:195–204.

[12] Kann O, Papageorgiou IE, Draguhn A. Highly energized inhibitory interneurons are a central element for information processing in cortical networks. J Cereb Blood Flow Metab. 2014;34:1270–1282. 24896567 10.1038/jcbfm.2014.104 PMC4126088

[13] Farrell JS, Gaxiola-Valdez I, Wolff MD, et al Postictal behavioural impairments are due to a severe prolonged hypoperfusion/hypoxia event that is COX-2 dependent. eLife. 2016;5:e19352. 27874832 10.7554/eLife.19352 PMC5154758

[14] Farrell JS, Colangeli R, Wolff MD, et al Postictal hypoperfusion/hypoxia provides the foundation for a unified theory of seizure-induced brain abnormalities and behavioral dysfunction. Epilepsia. 2017;58:1493–1501. 28632329 10.1111/epi.13827

[15] Robinson PA, Rennie CJ, Rowe DL. Dynamics of large-scale brain activity in normal arousal states and epileptic seizures. Phys Rev E Stat Nonlin Soft Matter Phys. 2002;65:041924. 12005890 10.1103/PhysRevE.65.041924

[16] Abeysuriya RG, Robinson PA. Real-time automated EEG tracking of brain states using neural field theory. J Neurosci Methods. 2016;258:28–45. 26523766 10.1016/j.jneumeth.2015.09.026

[17] Stuiver S, Tewarie PK, Pottkämper JCM, et al Temporal dynamics of electroconvulsive therapy induced seizures. Clin Neurophysiol. 2026;181:2111439. 41252922 10.1016/j.clinph.2025.2111439

[18] American Psychiatric Association . Diagnostic and statistical manual of mental disorders (5th ed.). American Psychiatric Association; 2013;105-168.

[19] Verdijk JPAJ, Pottkämper JCM, Verwijk E, et al Study of effect of nimodipine and Acetaminophen on postictal symptoms in depressed patients after electroconvulsive therapy (SYNAPSE). Trials. 2022;23:1–15. 35436940 10.1186/s13063-022-06206-y PMC9014277

[20] Pottkämper JCM, Verdijk JPAJ, Stuiver S, et al Exploring postictal recovery with Acetaminophen or nimodipine: A randomized-controlled crossover trial. Ann Clin Transl Neurol. 2024;11:2089–2300. 10.1002/acn3.52143 PMC11537141 39161097

[21] van den Broek WW, Birkenhäger T, de Boer D, et al Richtlijn Elektroconvulsietherapie. Nederlandse Vereniging voor Psychiatrie. In: Clinical guideline electroconvulsive therapy. Netherlands Association of Psychiatry. De Tijdstroom; 2010;70-90.

[22] d’Elia G, Ottosson JO, Strömgren LS. Present practice of electroconvulsive therapy in Scandinavia. Arch Gen Psychiatry. 1983;40:577–581. 6838335 10.1001/archpsyc.1983.01790050103013

[23] Sobin C, Sackeim HA, Prudic J, Devanand DP, Moody BJ, McElhiney MC. Predictors of retrograde amnesia following ECT. Am J Psychiatry. 1995;152:995–1001. 7793470 10.1176/ajp.152.7.995

[24] Rennie CJ, Robinson PA, Wright JJ. Effects of local feedback on dispersion of electrical waves in the cerebral cortex. Phys Rev E. 1999;59:3320–3329.

[25] Welch PD . The use of fast Fourier transform for the estimation of power spectra: A method based on time averaging over short, modified periodograms. IEEE Trans Audio Electroacoust. 1967;15:70–73.

[26] Akaike H . A new look at the statistical model identification. IEEE Trans Automat Contr. 1974;19:716–723.

[27] Bürkner PC . Brms: An R package for Bayesian multilevel models using Stan. J Stat Softw. 2017;80:1–28.

[28] Bürkner PC . Advanced Bayesian multilevel modeling with the R package brms. R J. 2018;10:395–411.

[29] Bürkner PC . Bayesian item response modeling in R with brms and Stan. J Stat Softw. 2021;100:1–54.

[30] Cassidy RM, Gale K. Mediodorsal thalamus plays a critical role in the development of limbic motor seizures. J Neurosci. 1998;18:9002–9009. 9787005 10.1523/JNEUROSCI.18-21-09002.1998 PMC6793529

[31] Lindquist BE, Timbie C, Voskobiynyk Y, Paz JT. Thalamocortical circuits in generalized epilepsy: Pathophysiologic mechanisms and therapeutic targets. Neurobiol Dis. 2023;181:106094. 36990364 10.1016/j.nbd.2023.106094 PMC10192143

[32] Hofmeijer J, Van Putten MJAM. Ischemic cerebral damage: An appraisal of synaptic failure. Stroke. 2012;43:607–615. 22207505 10.1161/STROKEAHA.111.632943

[33] Pottkämper JCM, Verdijk JPAJ, Aalbregt E, et al Changes in postictal cerebral perfusion are related to the duration of electroconvulsive therapy-induced seizures. Epilepsia. 2024;65:177–189. 37973611 10.1111/epi.17831

[34] Szubski C, Burtscher M, Löscher WN. The effects of short-term hypoxia on motor cortex excitability and neuromuscular activation. J Appl Physiol. 2006;101:1673–1677. 16902059 10.1152/japplphysiol.00617.2006

[35] Tewarie PK, Tjepkema-Cloostermans MC, Abeysuriya RG, Hofmeijer J, van Putten MJAM. Preservation of thalamocortical circuitry is essential for good recovery after cardiac arrest. PNAS Nexus. 2023;2:1–8. 10.1093/pnasnexus/pgad119 PMC10153639 37143862

[36] Johnston MV, Trescher WH, Ishida A, Nakajima W, Zipursky A. The developing nervous system: A series of review articles: Neurobiology of hypoxic-ischemic injury in the developing brain. Pediatr Res. 2001;49:735–741. 11385130 10.1203/00006450-200106000-00003

[37] Polyakov D, Robinson PA, Müßller EJ, et al Personalized stimulation therapies for disorders of consciousness: A computational approach to inducing healthy-like brain activity based on neural field theory. J Neural Eng. 2025;22:036033. 10.1088/1741-2552/addd48 40425026

[38] Sanders RD, Tononi G, Laureys S, Sleigh JW. Unresponsiveness ≠ unconsciousness. Anesthesiology. 2012;116:946–959. 22314293 10.1097/ALN.0b013e318249d0a7 PMC3311716

[39] Bodien YG, Allanson J, Cardone P, et al Cognitive motor dissociation in disorders of consciousness. N Engl J Med. 2024;391:598–608. 39141852 10.1056/NEJMoa2400645 PMC7617195

[40] Giese JL, Stanley TH. Etomidate: A new intravenous anesthetic induction agent. Pharmacother J Hum Pharmacol Drug Ther. 1983;3:251–258. 10.1002/j.1875-9114.1983.tb03266.x 6359080

