# Supplementary material for: Corticothalamic dynamics during postictal recovery of self-orientation after electroconvulsive therapy
Source: Brain Commun. 2026 Apr 22;8(3):fcag144. doi: 10.1093/braincomms/fcag144 (PMC13152015; doi:10.1093/braincomms/fcag144)
Supplement: fcag144_Supplementary_Data [file fcag144_supplementary_data.pdf]

Supplementary material

Gain parameters

Model gain parameters quantify the responsiveness of a neural population to synaptic input. They are defined as the product of the sensitivity of the population's firing rate to changes in its membrane potential and the synaptic coupling strength between populations. In our model, the firing rate of population a is given by

$$Q_a(t) = S(V_a(t)), \quad \text{(S1)}$$

where S is a sigmoidal function that converts the membrane potential $V_a(t)$ (in mV) into a firing rate (in Hz). The effective gain from population b to a is then defined as

$$G_{ab} = S'(V_a(t))\mu_{ab}, \quad \text{(S2)}$$

where $S'(V_a(t)) = \frac{dS(V)}{dV}\Big|_{V=V_a(t)}$ is the instantaneous slope (i.e. sensitivity) of the sigmoid at the current membrane potential and μ_{ab} is the coupling strength from population b to a, given by

$$\mu_{ab} = N_{ab}s_{ab} \quad \text{(S3)}$$

where N_{ab} is the number of synaptic connections and s_{ab} is the postsynaptic response per connection, expressed in mV/Hz. Gain parameters G_{ab} are thus dimensionless and reflect the product of synaptic efficacy and population excitability. Changes in gain can arise from (1) a shift in the operating point that alters the slope $S'(V_a)$, (2) changes in synaptic coupling strength μ_{ab}, or (3) a combination of both. For instance, an increase in gain may reflect a steeper firing-rate response curve, stronger synaptic feedback, or both.

Model fits to determine postictal temporal evolutions of model parameters

The results of the best-fitting model to determine the temporal behaviour of model parameters during the postictal state are shown in Supplementary Table 1.

Supplementary Table 1 Fitting models to determine postictal temporal behaviour of model parameters.

	Sat. exp. decay %	Sat. exp. growth %	Sigmoidal %
**Standard model gains**			
Cortical gain X	34.8	14.9	50.3
Corticothalamic loop gain Y	32.8	25.0	42.2
Intrathalamic gain Z	9.9	34.1	56
**Partial model gains**			
Cortical exc. feedback gain G_{ee}	55.7	31.8	12.5
Cortical inh. feedback gain G_{ei}	27.0	55.7	17.2
Corticothalamic gain G_{ct}	18.2	25.3	56.5
Thalamocortical gain G_{tc}	32.8	8.4	58.8
Thalamic inh. feedback gain G_{srs}	23.3	28.4	48.3

Sat.exp. = saturating exponential; exc. = excitatory; inh. = inhibitory

Posterior predictive checks

Posterior predictive checks for model 1 with parameters X (A), Y (B) and Z (C) and model 2 with G_{ee} (D), G_{ei} (E), G_{ct} (F), G_{tc} (G) and G_{srs} (H) indicate that the models provide adequate descriptions of the data. The observed values (dark blue line) fall well within the range of the replicated data (light blue lines), with no systematic discrepancies. This suggests that the models are compatible with the observed data.

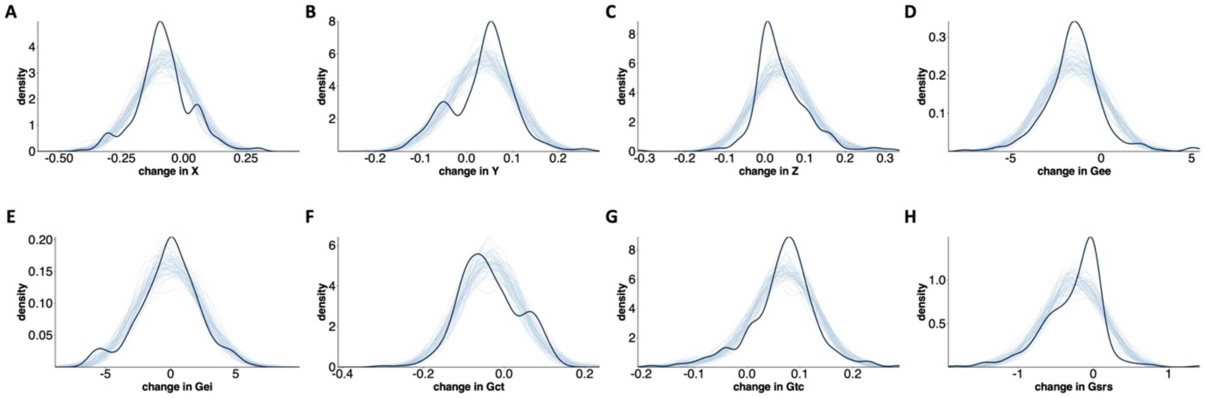

Supplementary Figure 1 Posterior predictive check plots of the Bayesian multivariate models. Model 1 consisted of the cortical gain *X* (A), corticothalamic loop gain *Y* (B) and intrathalamic gain *Z* (C). Model 2 consisted of the partial gains: cortical excitatory feedback G_{ee} (**D**), cortical inhibitory feedback G_{ei} (**E**), corticothalamic G_{ct} (**F**), thalamocortical G_{tc} (**G**) and thalamic inhibitory feedback gain G_{srs} (**H**). The dark blue line represents the observed data, while the light blue lines represent simulated responses drawn from the posterior predictive distribution of each model.

Model parameter estimation

Averaged goodness-of-fit (GOF) values of the model parameters estimation during the postictal state are shown in Supplementary Figure 2. GOF values showed no temporal effects during the postictal state, suggesting the model prediction accuracy was constant.

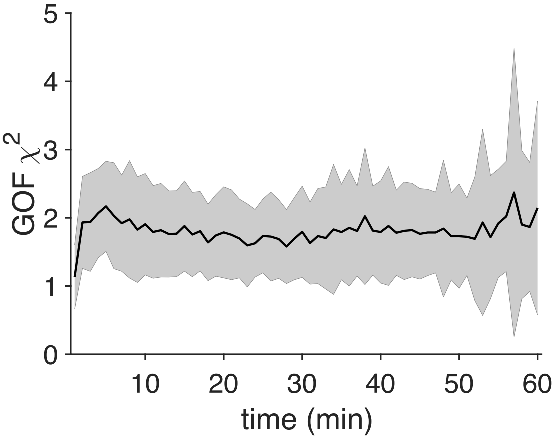

Supplementary Figure 2 Averaged goodness-of-fit (GOF) values of model parameter estimations during the postictal state. The solid line and shaded area represent the mean and standard deviation, respectively. Values are based on N = 345 postictal electroencephalography (EEG) recordings from n = 33 patients.
